# To: Foundational principles for young intensivists to drive better outcomes: the bedside application of physiology

**DOI:** 10.62675/2965-2774.20260054

**Published:** 2026-05-22

**Authors:** Anand Vinaykumar Joshi

**Affiliations:** 1 Mater Hospital Pimlico Intensive Care Unit Pimlico Australia Intensive Care Unit, Mater Hospital Pimlico - Pimlico - Australia.

DEAR EDITOR,

Rocco et al. cogently argue that a renewed focus on physiological reasoning is essential to advancing intensive care medicine and moving beyond rigid, protocol-driven practice.^(1)^ I agree with their central message but wish to extend it by highlighting an apparent paradox: almost everything we do in the intensive care unit (ICU) is, by necessity, profoundly nonphysiological. Our daily work is less an attempt to restore "normal" human physiology in real time than to purchase time – stabilizing patients with artificial supports while disease-modifying therapies take effect, and intrinsic reparative mechanisms attempt to re-establish a new equilibrium.

In routine ICU practice, we intentionally override native neurophysiologic and homeostatic systems. In the central nervous system, sedation is often continuous, sometimes supplemented by neuromuscular blockade and enforced immobility for days, suppressing arousal, sleep-wake cycling, spontaneous movement, and higher cortical function. Cardiovascular management likewise departs from normal physiology: vasopressors and inotropes are used to maintain a narrowly defined mean arterial pressure and heart rate, "flattening" natural blood pressure variability and inferring intravascular volume from pressure-based surrogates rather than from the individual's unknown premorbid hemodynamic set-points. Respiratory care is similarly artificial: evolutionary negative-pressure breathing is replaced by positive-pressure ventilation; fixed ventilatory patterns with limited variability are imposed; and uniform oxygenation and carbon dioxide ranges are targeted, which may not reflect chronic adaptation in patients with long-standing lung disease.^(2)^

Gastrointestinal and renal interventions further illustrate how far ICU care diverges from normal physiology. Continuous liquid enteral feeding bypasses the oral cavity and normal meal timing; proton-pump inhibitors blunt gastric acid secretion; prokinetics are used to enforce motility; and total parenteral nutrition circumvents the gut lumen and its complex enzymatic and neuroendocrine signaling. Renal and urinary management typically involves continuous bladder drainage for hourly urine output measurements, along with renal replacement therapy to manipulate urea, creatinine, and fluid balance, in contrast to the kidney's inherently pulsatile handling of solutes and water. Even fever, an evolutionarily conserved host defense, is frequently treated primarily as a vital-sign aberration, despite data suggesting potential immunologic benefit in selected contexts.^(2)^

Against this backdrop, the call for "physiology-guided" care requires clarification. Textbook physiological ranges are population-based reference intervals; by design, a substantial minority of healthy individuals fall outside them, and even within these limits, we rarely know any given patient's baseline. Translating such norms into rigid therapeutic targets risks reifying group-level data into individual prescriptions. For example, standard weaning and extubation criteria for an intubated patient with advanced chronic obstructive pulmonary disease often assume near-normal gas exchange. In contrast, that patient's pre-illness partial pressure of arterial oxygen (PaO_2_) and arterial partial pressure of carbon dioxide (PaCO_2_) may have been chronically "abnormal." The clinically relevant question is whether we can safely return the patient to their personal baseline, not whether we can normalize every variable.

Rocco et al.^(1)^ emphasize driving-pressure-targeted ventilation, dynamic assessment of fluid responsiveness, and tuning ventilatory settings to cerebral perfusion in specific scenarios. Building on this, I suggest distinguishing three layers of "physiology" at the bedside: understanding normal physiology to recognize patterns of compensation and decompensation; appreciating how critical illness distorts those patterns; and, most challenging, approximating each patient's baseline physiology as the true goal of individualization. Achieving this third layer requires inputs often absent from ICU workflows: collateral information from primary-care physicians or long-term specialists, detailed pre-illness history, and structured conversations with families about function and "usual" vitals. As Rocco et al.^(1)^ note, emerging tools such as integrated data systems and medical digital twins may eventually help simulate individualized responses and suggest safer, person-specific targets. However, their utility will depend on the fidelity of the underlying longitudinal data.

Protocols and guidelines, derived from the same physiological ranges, have undoubtedly improved outcomes yet can become quasi-gospels that constrain care within narrow, population-based boundaries. Simply substituting textbook "physiological" values for protocol targets does not constitute genuine personalization. If we truly seek individualized therapy, the gold standard should evolve toward approximating each patient's own physiological milieu, accepting that in chronic cardiopulmonary or renal disease, "success" may mean a return to a compensated, non-normal state.

By explicitly acknowledging the fundamentally nonphysiological nature of ICU practice, we can sharpen the role of physiology at the bedside: not to compel normalization of every parameter, but to help us distinguish adaptive compensation from irreversible organ failure and to steer artificial supports as close as is safely feasible to each patient's unique baseline.
